# Bacterial Species and Antimicrobial Resistance of Clinical Isolates from Pediatric Patients in Yangon, Myanmar, 2020

**DOI:** 10.3390/idr14010004

**Published:** 2022-01-06

**Authors:** Thida San, Meiji Soe Aung, Nilar San, Myat Myint Zu Aung, Win Lei Yi Mon, Thin Ei Thazin, Nobumichi Kobayashi

**Affiliations:** 1 Department of Clinical Laboratory, Yangon Children’s Hospital, Yangon 11191, Myanmar; drthidarsan@gmail.com (T.S.); thinei93@gmail.com (T.E.T.); 2 Department of Hygiene, School of Medicine, Sapporo Medical University, Sapporo 060-8556, Japan; meijisoeaung@sapmed.ac.jp; 3 Department of Microbiology, University of Medicine 2, Yangon 11031, Myanmar; drnilarsanum2@gmail.com; 4 Department of Microbiology, University of Medicine 1, Yangon 11131, Myanmar; myatmyintzuaung11@gmail.com; 5 Department of Clinical Laboratory, Yangon General Hospital, Yangon 11131, Myanmar; drwlym@gmail.com

**Keywords:** children, antimicrobial resistance, *Escherichia coli*, *Klebsiella pneumoniae*, MRSA, *Enterococcus*, ESBL, carbapenem, Myanmar

## Abstract

Antimicrobial resistance (AMR) is a concern in medical care for children who have high burden of infectious diseases. We investigated the prevalence of bacterial species and their susceptibility to antimicrobials of 1019 clinical isolates from pediatric patients in a tertiary-care hospital in Yangon, Myanmar for one-year period (2020). The most frequently recovered species was *Escherichia coli*, followed by *Klebsiella pneumoniae* and *Staphylococcus aureus*, all of which accounted for 43% of clinical isolates, while 25% of isolates comprised non-fermenter, including *Pseudomonas* sp. and *Acinetobacter* sp. Phenotypically determined ESBL (extended-spectrum beta-lactamase)-positive rates in *E. coli*, *K. pneumoniae*, and *Enterobacter* sp. were 82%, 88%, and 65%, respectively. High rates of multiple drug resistance were noted for *E. coli* (84%), *K. pneumoniae* (81%), and *Acinetobacter* sp. (65%), associated with carbapenem resistance in 48%, 42%, and 59% of isolates, respectively. In contrast, *S. aureus* isolates exhibited low resistance rates (<30%) to most of antimicrobials, with 22% being resistant to oxacillin/cefoxitin. Fluoroquinolone resistance was found in most of bacterial species with different prevalence rates. The present study revealed the current status on prevalence of bacterial species causing infections in pediatric patients in Myanmar, highlighting the significance to monitor AMR among children.

## 1. Introduction

Antimicrobial resistance (AMR) of pathogenic microorganisms is one of the major public health concerns. Southeast Asia has been regarded as a global hotspot of the emergence and spread of AMR because of increased antimicrobial demand and usage. This situation is considered to be implicated in population and economic growth, suboptimal knowledge and prescribing practice of antimicrobials, and also agriculture and aquaculture dependent on antimicrobials [1]. Particularly in low- and middle-income countries (LMICs), AMR is predicted to cause health and economic impact due to high burden of infectious diseases [2]; accordingly, surveillance to obtain accurate information on AMR is critical to proceed with appropriate treatment and reduction of AMR rates. However, comprehensive data of AMR are limited due to the limit of resources and technical capacity in these countries [2,3]. 

Clinically important bacterial groups/species that require rigid epidemiological surveillance of AMR include *Enterobacterales*, *Pseudomonas aeruginosa*, *Acinetobacter baumannii*, *Staphylococcus aureus*, and *Enterococcus faecium*, which are part of the “priority pathogen list” presented by WHO in 2007 [4]. In Asian regions, resistance to cephalosporins and carbapenems mediated by extended-spectrum beta-lactamases (ESBLs) or carbapenemases has been widespread among *Enterobacterales,* and other gram-negative rods belonging to non-fermenter [5]. Methicillin-resistant *Staphylococcus aureus* (MRSA) has been persistently prevalent, associated with spread of the regional and pandemic clones [6]. Investigation on relevance of these drug-resistant bacteria has been mainly focused on the adult population, while the information in children is limited [7,8,9]. Children are vulnerable to various infectious diseases, with bacteremia remaining a main cause of death in resource-poor settings; therefore, their optimal treatment is threatened by AMR [7].

In Myanmar, prevalence of pathogenic bacterial species and their drug resistance was studied mostly for adult patients [10,11,12,13,14]. Although the information of bacterial isolates from pediatric patients is available in a few studies on *Escherichia coli*, *S. aureus*, and neonatal sepsis [15,16,17], there is no comprehensive data on drug resistance in all the species of clinical isolates from children. In the present report, we describe the prevalence and drug resistance rates of all the clinical isolates from pediatric patients in a tertiary-care and teaching hospital in Yangon, Myanmar, during a one-year period to reveal characteristics and trend of current AMR situation in children.

## 2. Materials and Methods 

We conducted a cross-sectional, observational study in Yangon Children’s Hospital (YCH), which is a 550-bed hospital providing medical services to the pediatric population throughout the country. From January 2020 to December 2020, all the sequential clinical specimens from pediatric patients (blood, sputum, tracheal aspirate, urine, and pus/wound swabs, etc.) submitted to the Microbiology Laboratory of YCH for routine culture of pathogenic bacteria and their susceptibility testing were included in this study.

Each specimen was inoculated on sheep blood agar and MacConkey agar plates and incubated at 37°C overnight. Blood agar plates for sputum, wound swab, and blood/ CSF (cerebrospinal fluid) culture samples were incubated at 3–5% CO_2_ incubator. Bacterial culture from blood and CSF was initially performed by using BACTEC™ FX40 Instrument (Becton Dickinson, Sparks, NV, USA). Bacterial isolates from specimens were first detected by colonial morphology on agar plates and observed by ordinary light microscope following Gram staining. Identification and antibiotic susceptibility testing was performed by the Automated Microbiology System, BD Phoenix (Becton Dickinson, Sparks, NV, USA). In this system, a panel of 51 micro-wells containing substrates was used for bacterial identification. For antimicrobial susceptibility testing, a panel of 85 micro-wells was utilized, followed by bacterial growth detection by the use of a redox indicator (colorimetric oxidation-reduction). Resistance to antimicrobials for individual species of bacteria was judged in accordance with breakpoints mentioned in the Clinical and Laboratory Standards Institute (CLSI) guidelines (2019) [18]. ESBL production was phenotypically confirmed by using the BD Phoenix panel containing third-generation cephalosporin and third-generation cephalosporin plus clavulanic acid. In the present report, multiple drug resistance (MDR) was defined as resistance to three or more different classes of antimicrobials.

## 3. Results

During the one-year period, a total of 1019 isolates were recovered from 4133 clinical specimens submitted to the microbiology laboratory. Among the 23 different specimen types, blood was the most common (2036, 49%), followed by urine (1074, 26%), wound/pus (327, 8%), and CSF (267, 7%) (Table S1). Among the common specimens, the culture-positive rate of isolates was high (>60%) in wound/pus, ear swab, and tracheal aspirate, while blood showed a lower rate (14%).

Bacterial species identified in major clinical specimens and clinical wards are summarized in Table 1 and Table S2. The most frequently recovered species was *E. coli*, followed by *Klebsiella pneumoniae* and *S. aureus*, all of which accounted for 43% of the clinical isolates. In addition, 25% of isolates comprised non-fermenters including *Pseudomonas* sp. and *Acinetobacter* sp. From urine, *E. coli*, *K. pneumoniae*, and *Enterococcus* sp. were the most common (61% of all the urine isolates). Various bacterial species were identified from blood, including common ones, such as *K. pneumoniae*, *Enterobacter* sp., *Burkholderia cepacia*, *Acinetobacter* sp., *S. aureus,* and coagulase-negative staphylococci (CoNS). The major single-pathogenic species in wound/pus/tissue was *S. aureus* (38%), while the almost half of isolates were classified into gram-negative bacteria of various species. *P. aeruginosa*, *B. cepacia*, and *A. baumannii* were commonly identified for isolates from the respiratory specimens, accounting for 61%. Among the clinical wards in YCH, the highest number of isolates with various species were derived from the medical ward and the surgical ward (Table S2). In contrast, isolation rates of *Klebsiella* sp. and *S. aureus* were distinctively higher in the neonatal ward and the orthopedic ward, respectively.

**Figure 1:**
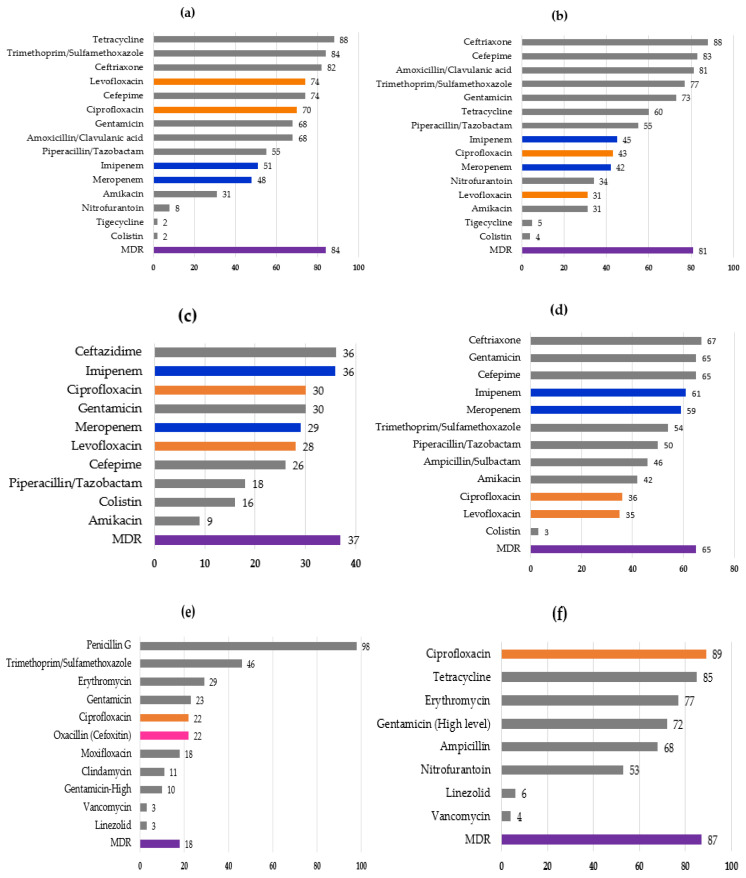
Resistance rates (%) to antimicrobials in six major species (genus) of bacteria ((**a**) *Escherichia coli*, (**b**) *Klebsiella* sp., (**c**) *Pseudomonas* sp., (**d**) *Acinetobacter* sp., (**e**) *Staphylococcus aureus*, (**f**) *Enterococcus* sp.) isolated in Yangon Children’s Hospital in 2020. The bars representing rates for quinolones, carbapenems, and oxacillin (cefoxitin) are shown in orange, blue, and pink, respectively. MDR represents multiple drug resistance, which was defined as resistance to three or more antimicrobials of different classes. For (**b**,**c**), MDR rates of *K. pneumoniae* and *P. aeruginosa* are shown, respectively. “Gentamicin—high” (**e**) and “Gentamicin (high level)” (**f**) represent MIC of ≥500 μg/mL. To assign resistance to tigecycline, interpretive criteria for *Enterobacterales* (MIC of 8 μg/mL or higher) defined by the U.S. Food and Drug Administration was employed.

Antimicrobial resistance rates for major bacterial group/species are shown in Figure 1a–f. Phenotypically determined ESBL-positive rates were 82%, 88%, and 65% in *E. coli*, *K. pneumoniae*, and *Enterobacter* sp., respectively. *E. coli* and *K. pneumoniae* exhibited >70~80% resistance rates to ceftriaxone and cefepime, and >40% of these species were resistant to carbapenems. More than half of *E. coli* and *K. pneumoniae* isolates were resistant to tetracycline, gentamicin, quinolones, and trimethoprim/sulfamethoxazole, showing MDR rates of 84% and 81%, respectively. *Acinetobacter* sp. showed generally higher resistance rates to most of antimicrobials examined than *Pseudomonas* sp., with higher rate of MDR (65%) than that of *Pseudomonas* sp. (37%) (Figure 1). *Enterobacter* sp. also showed a high MDR rate (61%). Only a few isolates of *E. coli*, *Klebsiella* sp., and *Acinetobacter* sp. (<5%) were resistant to colistin and tigecycline. 

*S. aureus* exhibited <30% resistance rates to most antimicrobials, except for penicillin G and trimethoprim/sulfamethoxazole. MRSA, which was defined by resistance to oxacillin/cefoxitin, accounted for 22% of *S. aureus*, and *Enterococcus* sp. showed high resistance rates (68–89%) to ampicillin, gentamicin (high-level resistance), erythromycin, tetracycline, and ciprofloxacin, while most isolates were susceptible to vancomycin and linezolid. Despite being variable in frequency, resistance to fluoroquinolones was commonly found in all the species, with *E. coli* and *Enterococcus* sp. showing high resistance rates (>70%).

## 4. Discussion

In the present report, a whole picture of bacterial isolates from pediatric patients and their resistance profiles was first described in Myanmar, in a tertiary-care hospital specialized for pediatric care. On the whole, major bacterial species from pediatric patients were *E. coli*, *K. pneumoniae*, *S. aureus*, and non-fermenter, represented by *Pseudomonas* sp. and *Acinetobacter* sp. A notable finding was the high rate of ESBL producers and carbapenem resistance in *Enterobacterales*. Resistance rate to ceftriaxone in *E. coli* and *K. pneumoniae* in the present study (82% and 88%, respectively) were comparable to or slightly higher than those reported for isolates from bacteremia/sepsis in Asian LMICs [2,7]. Previous reports in Myanmar described lower rates of ceftriaxone resistance in *E. coli* blood isolates (30%) [10], ESBL-positive rate among all the *E. coli* clinical isolates (67%) [11], and *K. pneumoniae* isolates from respiratory infections (37%) [14]. In contrast, *K. pneumoniae* from neonatal sepsis during 2017–2019 showed 93% resistance to ceftazidime [16], and our previous study in YCH in 2019 revealed that 81% of *E. coli* was phenotypically judged as an ESBL producer [17]. These findings suggest that ESBL has been highly prevalent among *E. coli* and *K. pneumoniae* in pediatric patients in Myanmar. 

The resistance rate to carbapenem of *K. pneumoniae* in the present study (42% to meropenem) appears to be similar to those in South Asian countries (40–46%), while it is higher than those in Southeast Asian countries (0–24%) [2] and also that reported for neonatal sepsis in Myanmar (12%) [16]. Among *E. coli*, carbapenem-resistance rate in clinical isolates from all the specimens in Myanmar was shown as 8.2% previously [11], in contrast to 48% in the present study. Accordingly, carbapenem resistance in *Enterobacterales* might have considerably spread over pediatric patients in Myanmar, which is of particular concern for *K. pneumoniae* because it is a major bacterial species from blood in neonates. Similarly, carbapenem resistance rates in *Pseudomonas* sp. (29%) and *Acinetobacter* sp. (59%) in our study were comparable to or higher than those in other Asian countries [2], indicating the need for continuous monitoring. In YCH, tigecycline is administered only for sepsis with *Enterobacterales* showing MDR, while colistin is not used. However, resistance to colistin or tigecycline was detected in some Gram-negative bacterial species despite low rate. In an adult patient with urinary tract infection, isolation of colistin-resistant *E. coli* harboring *mcr-1* has been reported in Myanmar [19]. Accordingly, monitoring of susceptibility to these last-resort drugs may be also necessary for pediatric isolates. 

Prevalence of MRSA among *S. aureus* (22%) was relatively low, which was similar to those in our previous study in YCH (19.7%) [15] as well as those in a tertiary-care hospital in Yangon (8–13.8%) [13,20]. *S. aureus* isolates from pediatric patients showed generally susceptibility to most antimicrobials, suggesting that drug resistance of *S. aureus* has not notably progressed in children. However, another report from the national AMR surveillance in Myanmar indicated higher prevalence of MRSA (48%) [21]. In addition, *S. aureus* isolates in Myanmar frequently carry Panton–Valentine leukocidin (PVL) genes [13,15,20], with significantly higher rate (68%) in methicillin-susceptible *S. aureus* from children [15]. Accordingly, the trend of prevalence of MRSA and virulence factors, including PVL, may be noted for future issues. In contrast, enterococcal species were highly resistant (>70%) to different classes of antimicrobials and was also associated with considerable resistance rate to nitrofurantoin (53%), which is commonly used for urinary tract infections. Although high susceptibility rates to vancomycin and linezolid seem to be maintained, further attention may be necessary to *Staphylococcus* and *Enterococcus* along with accurate evaluating measures to determine nonsusceptibility to these antimicrobials. 

It was of note in the present study that fluoroquinolone resistance was observed in different bacterial species, ranging from lower rates (22% in *S. aureus*) to high rates (74% in *E. coli*, 89% in *Enterococcus* sp.), although fluoroquinolones are not administered to pediatric patients except for older children. High rates of fluoroquinolone resistance in clinical isolates from children have been reported for *E. coli* in other studies [22,23]. It was postulated that the presence of fluoroquinolone resistance among children may be due to cross-infection of the resistant bacteria from adults or transmission of resistance genes associated with other antimicrobial resistance determinants colocalized on a same plasmid [23,24]. The different resistance rates to quinolones depending on bacterial species found in our present study may imply the presence of different genetic mechanisms of resistance, which remains to be elucidated.

During the present study, a surge of COVID-19 occurred in Myanmar from September to December 2020. However, in this period, only about 50 children with COVID-19 were admitted to YCH, and most of them had mild symptoms. Therefore, it seems that COVID-19 did not influence prevalence of bacterial species and AMR. 

The present report revealed the concerns for AMR in current pediatric isolates in Myanmar, i.e., high resistance rates to cephalosporin and carbapenem in *Enterobacterales* and non-fermenters and increased MDR trait in *Enterobacterales* and *Enterococcus* sp., in contrast to relatively low prevalence of MRSA. These findings highlighted the significance of resistance monitoring in pediatric isolates and also investigation for underlying resistance mechanisms to promote various measures to control AMR. In Myanmar, pediatricians use the guideline of antimicrobial use prepared by the Myanmar Pediatric Society. Considering the present situation of AMR as observed in this study, there may be a need to ensure the dissemination of the guideline along with any additional counterplan to strengthen antimicrobial policy.

## Figures and Tables

**Table 1:** Bacterial species/group recovered from major clinical specimens.

Bacterial Species	Number of Isolates in Each Specimen (%)					
	Urine	Blood	Wound/Pus/Tissue	Respiratory Specimens	Ear/Eye Discharge	Total
*Escherichia coli*	100 (32)	8 (3)	29 (13)			137 (15)
*Klebsiella* sp.	50 (16)	44 (16)	29 (13)	11 (13)		134 (14)
*Enterobacter cloacae*			6 (3)	4 (5)	3 (8)	13 (1)
other *Enterobacter* sp.		21 (7)				21 (2)
*Proteus mirabilis*					3 (8)	3 (0.3)
*Serratia marcescens*		11 (4)		3 (4)	2 (5)	16 (2)
*Salmonella* sp.		11 (4)				11 (1)
Other *Enterobacterales*	30 (10)	7 (2)	9 (4)		3 (8)	49 (6)
*Pseudomonas aeruginosa*	25 (8)		25 (12)	17 (20)	7 (19)	74 (8)
other *Pseudomonas* sp.		9 (3)				9 (1)
*Burkholderia cepacia*		38 (14)	1 (0.5)	18 (22)	1 (3)	58 (6)
*Stenotrophomonas maltophilia*				6 (7)		6 (0.6)
*Acinetobacter baumannii*			11 (5)	16 (19)	2 (5)	29 (3)
*Acinetobacter* sp.		17 (6)				17 (2)
Other non-fermenter	25 (8)	11 (4)		3 (4)		39 (4)
*Staphylococcus aureus*		19 (7)	81 (38)	4 (5)	9 (24)	113 (12)
Coagulase-negative *Staphylococci*	7(2)	23 (8)	17 (8)		4 (11)	51 (5)
*Streptococcus pyogenes*					1 (3)	1 (0.1)
*Streptococcus* sp.	2 (1)	4 (1)	4 (2)			10 (1)
*Enterococcus* sp.	40 (13)	4 (1)	2 (1)			46 (5)
*Candida* sp.	36 (11)	53 (19)	2 (1)	1 (1)	1 (3)	93 (10)
*Cryptococcus neoformans*		1 (0.4)				1 (0.1)
Yeast/mold					1 (3)	1 (0.1)
total	315 (100)	281 (100)	216 (100)	83 (100)	37 (100)	932 (100)

## Data Availability

Not applicable.

## Supplementary Materials

The following are available online at https://www.mdpi.com/article/10.3390/idr14010004/s1, Table S1: Number of isolated pathogens from each specimen in YCH, 2020, Table S2: Prevalence of bacterial species in each clinical ward in YCH, 2020.

## References

[1] Zellweger R.M., Carrique-Mas J., Limmathurotsakul D., Day N.P.J., Thwaites G.E., Baker S. (2017). Southeast Asia Antimicrobial Resistance Network. A current perspective on antimicrobial resistance in Southeast Asia. J. Antimicrob. Chemother..

[2] Gandra S., Alvarez-Uria G., Turner P., Joshi J., Limmathurotsakul D., van Doorn H.R. (2020). Antimicrobial Resistance Surveillance in Low- and Middle-Income Countries: Progress and Challenges in Eight South Asian and Southeast Asian Countries. Clin. Microbiol. Rev..

[3] Kakkar M., Chatterjee P., Chauhan A.S., Grace D., Lindahl J., Beeche A., Jing F., Chotinan S. (2018). Antimicrobial resistance in South East Asia: Time to ask the right questions. Glob. Health Action.

[4] World Health Organization Global Priority List of Antibiotic-Resistant Bacteria to Guide Research, Discovery, and Development of New Antibiotics. https://www.who.int/medicines/publications/WHO-PPL-Short_Summary_25Feb-ET_NM_WHO.pdf.

[5] Hawkey P.M. (2015). Multidrug-resistant Gram-negative bacteria: A product of globalization. J. Hosp. Infect..

[6] Chen C.J., Huang Y.C. (2014). New epidemiology of *Staphylococcus aureus* infection in Asia. Clin. Microbiol. Infect..

[7] Le Doare K., Bielicki J., Heath P.T., Sharland M. (2015). Systematic Review of Antibiotic Resistance Rates Among Gram-Negative Bacteria in Children with Sepsis in Resource-Limited Countries. J. Pediatric Infect. Dis. Soc..

[8] Versporten A., Bielicki J., Drapier N., Sharland M., Goossens H., ARPEC project group (2016). The Worldwide Antibiotic Resistance and Prescribing in European Children (ARPEC) point prevalence survey: Developing hospital-quality indicators of antibiotic prescribing for children. J. Antimicrob. Chemother..

[9] Fu P., Xu H., Jing C., Deng J., Wang H., Hua C., Chen Y., Chen X., Zhang T., Zhang H. (2021). Bacterial Epidemiology and Antimicrobial Resistance Profiles in Children Reported by the ISPED Program in China, 2016 to 2020. Microbiol. Spectr..

[10] Myat T.O., Hannaway R.F., Zin K.N., Htike W.W., Win K.K., Crump J.A., Murdoch D.R., Ussher J.E. (2017). ESBL- and Carbapenemase-Producing *Enterobacteriaceae* in Patients with Bacteremia, Yangon, Myanmar, 2014. Emerg. Infect. Dis..

[11] Aung M.S., San N., Maw W.W., San T., Urushibara N., Kawaguchiya M., Sumi A., Kobayashi N. (2018). Prevalence of Extended-Spectrum Beta-Lactamase and Carbapenemase Genes in Clinical Isolates of *Escherichia coli* in Myanmar: Dominance of *bla*_NDM-5_ and Emergence of *bla*_OXA-181_. Microb. Drug Resist..

[12] Myat T.O., Oo K.M., Mone H.K., Htike W.W., Biswas A., Hannaway R.F., Murdoch D.R., Ussher J.E., Crump J.A. (2020). A prospective study of bloodstream infections among febrile adolescents and adults attending Yangon General Hospital, Yangon, Myanmar. PLoS Negl. Trop. Dis..

[13] Aung M.S., San T., Urushibara N., San N., Oo W.M., Soe P.E., Kyaw Y., Ko P.M., Thu P.P., Hlaing M.S. (2020). Molecular Characterization of Methicillin-Susceptible and -Resistant *Staphylococcus aureus* Harboring Panton-Valentine Leukocidin-Encoding Bacteriophages in a Tertiary Care Hospital in Myanmar. Microb. Drug Resist..

[14] Aung M.S., Win N.C., San N., Hlaing M.S., Myint Y.Y., Thu P.P., Aung M.T., Yaa K.T., Maw W.W., Urushibara N. (2021). Prevalence of Extended-Spectrum Beta-Lactamase/Carbapenemase Genes and Quinolone-Resistance Determinants in *Klebsiella pneumoniae* Clinical Isolates from Respiratory Infections in Myanmar. Microb. Drug Resist..

[15] Aung M.S., San T., Urushibara N., San N., Hlaing M.S., Soe P.E., Htut W.H.W., Moe I., Mon W.L.Y., Chan Z.C.N. (2021). Clonal Diversity and Molecular Characteristics of Methicillin-Susceptible and -Resistant *Staphylococcus aureus* from Pediatric Patients in Myanmar. Microb. Drug Resist..

[16] Oo N.A.T., Edwards J.K., Pyakurel P., Thekkur P., Maung T.M., Aye N.S.S., New H.M. (2021). Neonatal Sepsis, Antibiotic Susceptibility Pattern, and Treatment Outcomes among Neonates Treated in Two Tertiary Care Hospitals of Yangon, Myanmar from 2017 to 2019. Trop. Med. Infect. Dis..

[17] San T., Moe I., Ashley E.A., San N. (2021). High burden of infections caused by ESBL-producing MDR *Escherichia coli* in paediatric patients, Yangon, Myanmar. JAC Antimicrob. Resist..

[18] Clinical and Laboratory Standards Institute (CLSI) (2019). Performance Standards for Antimicrobial Susceptibility Testing, 29th Informational Supplement, M100–S129.

[19] San N., Aung M.S., Thu P.P., Myint Y.Y., Aung M.T., San T., Mar T.T., Lwin M.M., Maw W.W., Hlaing M.S. (2019). First detection of the *mcr-1* colistin resistance gene in *Escherichia coli* from a patient with urinary tract infection in Myanmar. New Microbes New Infect..

[20] Aung M.S., Zi H., New K.M., Maw W.W., Aung M.T., Min W.W., Nyein N., Kawaguchiya M., Urushibara N., Sumi A. (2016). Drug resistance and genetic characteristics of clinical isolates of staphylococci in Myanmar: High prevalence of PVL among methicillin-susceptible *Staphylococcus aureus* belonging to various sequence types. New Microbes New Infect..

[21] Soe P.E., Han W.W., Sagili K.D., Satyanarayana S., Shrestha P., Htoon T.T., Tin H.H. (2021). High Prevalence of Methicillin-Resistant *Staphylococcus aureus* among Healthcare Facilities and Its Related Factors in Myanmar (2018–2019). Trop. Med. Infect. Dis..

[22] Logan L.K., Medernach R.L., Rispens J.R., Marshall S.H., Hujer A.M., Domitrovic T.N., Rudin S.D., Zheng X., Qureshi N.K., Konda S. (2019). Community Origins and Regional Differences Highlight Risk of Plasmid-mediated Fluoroquinolone Resistant Enterobacteriaceae Infections in Children. Pediatr. Infect. Dis. J..

[23] Akgoz M., Akman I., Ates A.B., Celik C., Keskin B., Ozmen Capin B.B., Karahan Z.C. (2020). Plasmidic Fluoroquinolone Resistance Genes in Fluoroquinolone-Resistant and/or Extended Spectrum Beta-Lactamase-Producing *Escherichia coli* Strains Isolated from Pediatric and Adult Patients Diagnosed with Urinary Tract Infection. Microb. Drug Resist..

[24] Abelson Storb K., Osterlund A., Kahlmeter G. (2004). Antimicrobial resistance in *Escherichia coli* in urine samples from children and adults: A 12 year analysis. Acta Paediatr..

