# Supplementary material for: Bacterial Species and Antimicrobial Resistance of Clinical Isolates from Pediatric Patients in Yangon, Myanmar, 2020
Source: Infect Dis Rep. 2022 Jan 6;14(1):26–32. doi: 10.3390/idr14010004 (PMC8788269; doi:10.3390/idr14010004)
Supplement: Supplementary file 1 [file idr-14-00004-s001.zip › Supplementary Tables S1and S2.pdf]

Table S1. Number of isolated pathogens from each specimen in YCH, 2020.

specimens	Total number of specimens	Number of isolated pathogens (%)
Blood	2036	281 (14)
Urine	1074	315 (29)
Wound and Pus	327	203 (62)
CSF	267	13 (5)
Ear swab	34	22 (65)
Tracheal aspirate	92	62 (67)
Intravenous Catheter tip	68	19 (28)
Eye swab	30	15 (50)
Throat swab	10	0
Sputum	40	19 (48)
Pleural fluid	11	2 (18)
Peritoneal dialysis	27	11 (41)
Tissue	25	13 (52)
Nasal swab	11	5 (45)
Stool and Rectal swab	52	28 (54)
Joint fluid	8	1 (13)
Skin swab	4	1 (25)
Ascitic fluid	4	2 (50)
Vagina swab	5	3 (60)
Bile	3	2 (67)
Drain	3	0
VP shunt tip	1	1 (100)
Pericardial fluid	1	1 (100)
total	4133	1019 (25)

Table S2. Prevalence of bacterial species in each clinical ward.

clinical ward	No. of isolates belonging to bacterial species															
	E.coli	*Klebsiella*	*Enterobacter*	*Proteus*	*Serratia*	*Salmonella*	Other *Entero-bacterales*	*Pseudomonas*	*Acinetobacter*	Other Non-fermenter	*S. aureus*	CoNS	*Enterococcus*	*Streptococcus*	Yeast & Mould	Total
Medical	66	41	8	6	0	11	11	16	17	14	21	13	15	5	20	264
Surgical	47	34	5	1	1	0	8	29	13	15	23	18	11	3	27	235
Renal	10	7	2	2	1	0	1	8	1	5	5	2	3	0	6	53
Haemato-oncology	15	4	4	0	0	0	0	4	2	5	2	6	2	1	7	52
Neonate	9	28	16	0	5	0	6	5	7	9	8	11	0	4	9	117
Neuro	2	1	0	0	0	0	0	0	0	0	0	0	1	0	0	4
ICU	2	7	0	1	0	0	0	14	10	27	2	0	0	0	6	69
SICU	6	12	7	0	13	0	0	8	14	22	3	1	3	0	31	120
Ortho	5	1	1	1	0	0	1	4	1	0	56	3	1	0	0	74
OPD	14	1	2	1	0	0	2	0	1	0	3	1	3	3	0	31
Total	176	136	45	12	20	11	29	88	66	97	123	55	39	16	106	1019
